# How to improve private ophthalmology services, a marketing perspective

**Published:** 2019

**Authors:** Consuela Mădălina Gheorghe

**Affiliations:** * Philologist, Authorized translator

At present, private ophthalmology services are regarded as very important for patients concerned about their sight health and whole wellbeing. In order to have healthy eyesight they tend to choose very carefully the private clinic they decide to go to for a consultation, prescription, or regular eye checkup. In addition, the private ophthalmology clinics managers have to think very well which is the best marketing technique they need to apply in order to improve the private ophthalmology services. 

What should be mentioned first is the marketing plan, which is tailored so that the visibility of the private ophthalmology clinic and its services are enhanced in the community. This will lead to a growth of the patient base. What is worth mentioning at this point is traditional marketing, such as branding and also print advertising and online marketing, here also including custom websites, search engine optimization and online reputation management. 

It is already known that the private ophthalmology clinics vary in size, structure, and services. It seems more obvious that practitioners and clinics are constantly challenged to attract patients who have the most serious cases, such as cataract and refractive eye problems that need surgery. However, attracting new patients and cases has turned out to be very difficult lately. At the same time, the need to aggressively promote the private ophthalmology clinic has become a constant business imperative. As a result, the need for marketing and advertising has become a must. 

When dealing with the dynamics of marketing and advertising we should take into account the following: the patient is an empowered consumer (meaning that the patient wishes to take part in his healing process, wishes to communicate and collaborate with the ophthalmologist regarding his illness and treatment, he searches the Internet regarding his illness, talks to his peers about his illness, etc.), professional referral sources are accessible (at a certain moment, a relatively steady stream of referrals from the optometrist, family physicians and other professionals provided an equally steady revenue stream, but, as healthcare reshaped that landscape, the flow of patients has been diverted or redirected to a less predictable course) (https://www.healthcaresuccess.com/blog/doctor-marketing/ophthalmology-marketing-plan.html), competition is smart and aggressive (in order to keep the existent patients and to win new ones, the private ophthalmology clinics need to appeal to marketing and advertising campaigns), private ophthalmology services have matured and the patients have become more price-sensitive (without the proper ophthalmology marketing plan, each clinic is common and it should be different in such a way that it attracts the patient and it makes him loyal, by implementing a fair quality-price rate of the services offered), reputation of a private ophthalmology clinic stands in the online marketing (since patients like to search the Internet for everything concerning their health, if a private clinic cannot be met online, it does not exist).

When creating a marketing plan for a private ophthalmology clinic, the following should be taken into account: shot-term objectives (less than one year), long-term objectives (one year or more), target audience, what does the target audience want, who you are, analyze competition, budget, how to compete, plan of execution https://www.reviewofophthalmology.com/article/getting-started-with-a-marketing-plan). 

The following steps need to be taken to refresh the private ophthalmology clinic-marketing plan (https://www.healthcaresuccess.com/blog/doctor-marketing/ophthalmology-marketing-to-boost-business.html): use an unbiased SWOT analysis – the daily demands of a private ophthalmology clinic can easily distract attention from the dynamics of change. But a high-level examination of Strengths, Weaknesses, Opportunities and Threats is the vital strategic planning step to refocus; look inside first – the internal audiences, meaning those people and patients who already know the private ophthalmology clinic – usually represent a low-barrier opportunity to reach a large and important audience; inform all the patients what you do – any given patient knows almost nothing about the full range of services, practice or procedures of any private ophthalmology clinic. The capabilities may seem obvious to the manager and staff of the private ophthalmology clinic, but the patient, or someone they know, may benefit from knowing more about the services offered; a private ophthalmology clinic representative inspires professional referrals – ophthalmology clinics that are referral dependent often remain a clinic reputation or physician liaison to protect and expand the income stream. Referring physicians – family or general practitioners – as well as optometrists, opticians and others is a valuable marketing and business resource.

In conclusion, a successful marketing and business development requires a commitment to achieve personal and professional goals. In order to win in today’s competitive ophthalmological environment requires a third party assessment and also experienced, professional support. 

**Assist. Prof. Consuela-Mădălina Gheorghe, PhD,** **Philologist, Authorized translator**

